# Construction and Application of Graphene Oxide-Bovine Serum Albumin Modified Extended Gate Field Effect Transistor Chiral Sensor

**DOI:** 10.3390/s21113921

**Published:** 2021-06-07

**Authors:** Le Li, Xiaofei Ma, Yin Xiao, Yong Wang

**Affiliations:** 1 Tianjin Key Laboratory of Molecular Optoelectronic Science, Department of Chemistry, School of Science, Tianjin University, Tianjin 300072, China; ll17865181937@163.com; 2 School of Chemical Engineering and Technology, Tianjin Engineering Research Center of Functional Fine Chemicals, Tianjin University, Tianjin 300072, China; xiaoyin@tju.edu.cn

**Keywords:** extended-gate field effect transistor, bovine serum albumin, graphene oxide, chiral sensing

## Abstract

Chirality is an essential natural attribute of organisms. Chiral molecules exhibit differences in biochemical processes, pharmacodynamics, and toxicological properties, and their enantioselective recognition plays an important role in explaining life science processes and guiding drug design. Herein, we developed an ultra-sensitive enantiomer recognition platform based on an extended-gate metal-oxide semiconductor field-effect-transistor (Nafion–GO@BSA–EG-MOSFET) that achieved effective chiral resolution of ultra-sensitive Lysine (Lys) and α-Methylbenzylamine (α-Met) enantiodiscrimination at the femtomole level. Bovine serum albumin (BSA) was immobilized on the surface of graphene oxide (GO) through amide bond coupling to prepare the GO@BSA complex. GO@BSA was drop-cast on deposited Au surfaces with a Nafion solution to afford the extended-gate sensing unit. Effective recognition of chiral enantiomers of mandelic acid (MA), tartaric acid (TA), tryptophan (Trp), Lys and α-Met was realized. Moreover, the introduction of GO reduced non-specific adsorption, and the chiral resolution concentration of α-Met reached the level of picomole in a 5-fold diluted fetal bovine serum (FBS). Finally, the chiral recognition mechanism of the as-fabricated sensor was proposed.

## 1. Introduction

Homochirality in nature is a mystery that plagues the scientific community [1]. Usually, chiral molecules in vivo participate in enantioselectivity, and the enantiomers of chiral molecules may exhibit completely different physiological activities and toxicological properties [2]. Therefore, recognizing chiral enantiomers is of great significance for drug design and understanding the metabolism and regulation in organisms. At present, the identification and resolution of chiral molecules are usually achieved by means of high-performance liquid chromatography [3], capillary electrophoresis [4], electrochemistry [5], and circular dichroism [6], which are often time-consuming, require cost-ineffective equipment, produce low responses, and present difficulty achieving real-time online analysis. Consequently, the development of a new type of enantiomer recognition platform is key to meeting the needs of label-free, highly sensitive, fast-response detection. Considering the above requirements, the attraction of field-effect transistors (FETs) for sensing is multidimensional [7]. In the detection of target molecules, the liquid environment of the analyte may cause irreversible damage to the performance of the device. The separation of the sensing unit and device by the extend-gate FET (EGFET) can effectively overcome defects to ensure device stability [8,9]. Field-effect transistors have been proven to be an ideal fast and sensitive sensing platform [10,11]. However, high-sensitivity chiral sensing still needs further exploration. Mulla et al. [12] measured the weak chiral interactions associated with neutral enantiomers and found that they differentially bind to odorant binding proteins (OBPs) by bio-FET. Iskierko et al. [13] used an extended-gate metal-oxide semiconductor field-effect-transistor (EG-MOSFET) with a detection limit of 13 μM for the enantioselective determination of phenylalanine isomers. The above research tells us that FET has great potential for chiral recognition.

Graphene oxide (GO) is a two-dimensional single sheet structure composed of carbon atoms and is increasingly recognized for its unique optoelectronic properties, mechanical robustness, and superior thermal stability [14]. GO has the advantages of enriched oxygen-containing functional groups––high electrical conductivity, large specific surface area, good biocompatibility, and high physiological stability [15]––which makes it conducive to the surface modification of biomolecules or nanomaterials, and its anti-interference performance in analyte detection suggests broad application prospects in biomedicine, analysis, sensing and other fields.

As a typical representative of natural chiral separation media, the enantiomer recognition ability of bovine serum albumin (BSA), has recently been verified in many fields [16,17,18]. The surface of BSA contains free amino groups that can be covalently connected to the surface of GO through amide bonds. By taking advantage of natural recognition sites, the unique multi-level structure of proteins, and the differences between chiral molecules in hydrophobic interaction, electrostatic interaction and hydrogen bonding give full play to BSA’s superior chiral recognition ability [19,20].

Inspired by the signal amplification and conduction characteristics of EG-MOSFET, the superior chiral recognition of BSA, and the anti-interference advantages of GO, an extended-gate field effect transistor sensor modified with graphene oxide-bovine serum albumin composite material (Nafion–GO@BSA-EG-MOSFET) was constructed, thereby furnishing an efficient research platform and detection technique for the rapid and sensitive distinction of chiral isomers and biomolecules.

## 2. Materials and Methods

### 2.1. Chemicals and Reagents

Graphene oxide was purchased from Zhongke Times Nanotech Co., Ltd. (Chengdu, China). Fetal bovine serum (FBS) was purchased from Yibo Hengtai Biotechnology Co., Ltd. (Tianjin, China). BSA, D-Tryptophan (D-Trp), L-Tryptophan (L-Trp), L-Lysine (L-Lys), D-Lysine (D-Lys), R-(+)-α-Methylbenzylamine (R-(+)-α-Met), S-(+)-α-Methylbenzylamine (S-(+)-α-Met) were from Aladdin Chemical Co., Ltd. (Shanghai, China). L-(−)-Mandelic acid (L-MA), D-(−)-Mandelic acid (L-MA), L-Tartaric acid (L-TA), D-Tartaric acid (D-TA), potassium chloride (KCl), Potassium ferricyanide (K_3_Fe(CN_6_), potassium ferrocyanide (K_4_Fe(CN)_6_) and other unmentioned chemicals all came from Heowns (Tianjin, China). All aqueous solutions were made from an ultra-pure water (Milli-Q, Millipore, Darmstadt, Germany) preparation. Polydimethylsiloxane (PDMS) was purchased from Dow Corning (Shanghai, China) Co., Ltd. Acetone, absolute ethanol and isopropanol were purchased from Rianlon Corporation (Tianjin, China). Phosphate-buffered saline (PBS, 10 mM) was purchased from Shanghai Beyotime Biotechnology Co., Ltd. (Shanghai, China). NMOS and glass slides were purchased locally. Nafion solution was purchased from DuPont. 

### 2.2. FTIR and UV-Vis Characterization

Fourier Transform infrared spectroscopy (FTIR, ALPHA, German BRUKER) characterized the raw materials and products with KBr as the background. The spectral range was 4000–500 cm^−1^, and the scanning speed was 10 times/s.

Ultraviolet-visible spectroscopy (UV-vis, UV-1800PC) was used to scan in the range of 400–200 nm with a scanning interval of 1 nm.

### 2.3. X-ray Photoelectron Spectroscopy

The PHI-5000 Versa probe (Kanagawa-ken, Japan) was used for XPS characterization of the GO, GO-COOH and GO@BSA.

### 2.4. Electrochemical Measurements

All electrochemical testing was performed by CHI 650E electrochemical workstation (Shanghai Chenhua Instruments Co., Shanghai, China). In the standard three-electrode system, the saturated calomel electrode was used as the reference electrode, the platinum wire electrode worked as the counter electrode, and the working electrode was gold-plated glass (GE, Au 1.0 × 1.0 cm) or modified gold-plated glass (Nafion–GO-GE, Nafion–GO@BSA-GE, Nafion–BSA-GE). The electrolyte solution contains 5 mM [Fe(CN)_6_]^4-/3-^ and 0.1 M KCl (pH 7).

Cyclic voltammogram (CV) parameters were set as follows: the scan interval ranged from –0.2 to +0.6 V, and the scan speed was 20 mV·s^−1^. The electrochemical impedance spectroscopy (EIS) measurement was under open-circuit voltage, amplitude was set to 5 mV, and the frequency range was 10^−2^–10^5^ Hz. Differential pulse voltammograms (DPV) were recorded in the potential range of –0.2 to +0.6 V at the default parameters.

### 2.5. Preparation of GO@BSA

**Figure 1:**
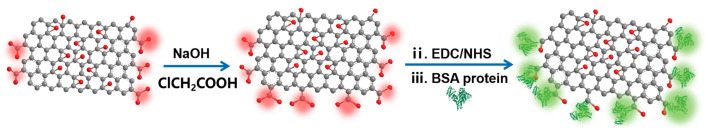
Preparation process of GO@BSA.

The preparation process of GO@BSA is shown in Figure 1. Graphene oxide powder was mixed with deionized water, and ultrasonic dispersion was carried out for 1 h to prepare a uniform solution with the concentration of 1 mg/mL. Then, NaOH and ClCH_2_COOH were slowly added several times under the condition of an ice bath and stirring. Ultrasonic treatment was applied for 2 h and then dilute hydrochloric acid (HCl) was used to wash the compound until the pH reached 7 to obtain GO–COOH [21].

EDC/NHS (1-ethyl-3-(3-dimethylaminopropyl) carbodiimide hydrochloride/N-hydroxysuccinimide) were dissolved in an MES buffer solution (pH 5), mixed with GO-COOH and activated for 40 min [22]. The excess EDC, NHS and byproducts were removed by repeated washing with a buffer solution. BSA was dissolved in a 0.01 M phosphate buffer (pH 7.2) and added to the activated GO-COOH. The amino group of BSA coupled with the carboxyl group of GO via an amide bond after 24 h of stirring. Finally, GO@BSA was obtained by repeated washing with a buffer solution and freeze-drying.

### 2.6. Immobilization of BSA, GO or GO@BSA on Au Electrode

Firstly, a gold film of 80 nm was deposited on a glass substrate by thermal evaporation. Then, the gold-plating substrate was cleaned with acetone, absolute ethanol, isopropanol, and deionized water.

1 mg/mL BSA, GO or GO@BSA was each blended with a 0.5%wt Nafion solution (*v/v*, 1:1), and the uniform dispersion system was formed by Lab dancer (IKA, Königswinter, Germany). The gold-plated glass was treated with Plasma at 80 W for 10 min to enhance the surface hydrophilicity. Then, 20 μL volume of Nafion–BSA, Nafion–GO or Nafion–GO@BSA suspension were drop-coated onto the surface of the gold-plated glass to form a uniform liquid film, which evaporated naturally at room temperature (Supplementary Figure S1).

### 2.7. FET Sensing Procedure

The electrical characteristics of field-effect transistors were measured under environmental conditions with a Keithley 2400 SCS. A wire clip was used to connect the extended gate and the MOSFET, and the gate voltage was applied through a copper wire.

For non-real-time detection, a sample solution was pipetted to a PDMS detection cell installed on the extended-gate electrode modified by the GO@BSA complex and allowed to stand for 30 s. The output characteristic curve of the chiral sensor was measured under the condition of V_GS_ = V_DS_ = 2 V.

During real-time detection, 1% PBS (0.1 mM) was added to the PDMS detection cell to establish a stable baseline. Then, the chiral enantiomer solution with 1% PBS buffer as the solvent was injected into the PDMS detection cell according to the concentration gradient. The time interval of each concentration was about 30 s.

## 3. Results and Discussion

### 3.1. Characterization of GO@BSA

**Figure 2:**
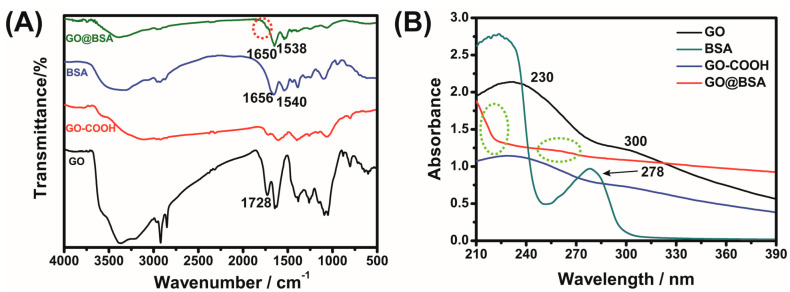
Fourier Transform infrared spectroscopy (FTIR) (**A**) and UV-vis (**B**) spectroscopy of GO, GO–COOH, BSA, GO@BSA.

FTIR spectroscopy was employed to examine the GO, BSA, GO-COOH and GO@BSA materials. As shown in Figure 2A, the characteristic BSA absorption peaks at 1656 cm^−1^ and 1540 cm^−1^ represent the amide I band and amide II band, respectively [18,23]. For GO [24], the broad and strong absorption peak near 3400 cm^−1^ belonged to O–H stretching vibration; the absorption peak at 2921 cm^−1^ was assigned to the C–H stretching vibration, and the absorption peak at 1728 cm^−1^ related to the C=O stretching vibration on the carboxyl group. The characteristic peak at 1500 and 1600 cm^−1^ referred to the benzene frame vibration on GO, and the characteristic peaks at 1050 and 1260 cm^−1^ corresponded to the stretching vibration of epoxy C–O–C. Compared with GO, the carboxylic acid peak of GO@BSA complex disappeared at 1728 cm^−1^, indicating that -COOH had reacted with -NH_2_ to form an amide bond, the stretching vibration peak of NH–CO at 1650 cm^−1^, and the bending vibration peak of N–H shifted from 1540 to 1538 cm^−1^, indicating a new amide bond between GO and BSA [23].

The experimental results of UV-vis spectroscopy are shown in Figure 2B. The characteristic peak of GO at 230 nm is related to the π → π* transition from aromatic C=C, and the shoulder peak at 300 nm was caused by the n → π* transition of C=O. The absorption peak of BSA at 278 nm was generated via the n → π* transition from aromatic amino acid residues. The UV-vis spectroscopy of GO did not change after carboxylation. However, the spectroscopy of the GO@BSA complex clearly changed, and terminal absorption appeared [25].

**Figure 3:**
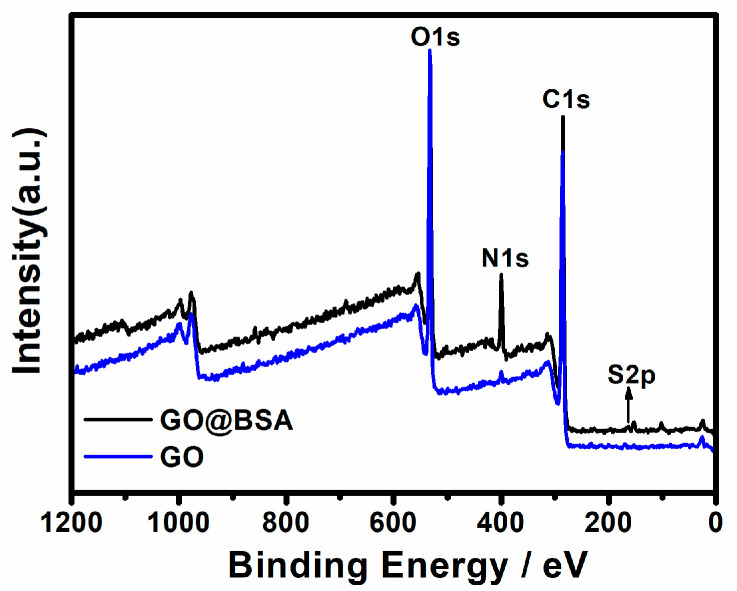
X-ray photoelectron spectroscopy (XPS) fully scanned spectra of GO and GO@BSA.

The element composition of GO and GO@BSA was discussed by XPS (Figure 3). For GO and BSA, the main elements were C, N, O and S. The N and S of GO originated from the introduction of impurities into the industrial production. Before and after the GO coupling protein, the content of C, N and O changed significantly. As summarized in Table 1, the content of C decreased by 2.61%; the content of N increased by 8.43%; and the content of O decreased by 5.53%. In addition, the O1s spectra and content [23,26] are presented in Supplementary Figure S2 and Table S1. The content comparison of O valence states indicated that the content of amide O increased significantly after the BSA coupling, and N mainly existed in the form of amide N and amino N.

In conclusion, combined with the FTIR and UV-vis spectroscopy, BSA was successfully coupled to GO.

### 3.2. Characterization of Sensitized Au Electrode

**Figure 4:**
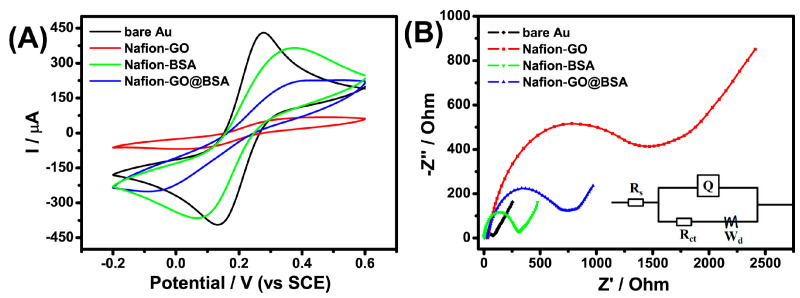
Cyclic voltammogram (CV) (**A**) and Nyquist plots (**B**) in the electrolyte containing 5 mM [Fe(CN)_6_]^4-/3-^ and 0.1 M KCl. Scan rate of CV, 20 mV·s^−1^; frequency range of Nyquist plots, 10^5^ to 10^−2^ Hz. B inset: Equivalent circuit.

CV and EIS were employed to evaluate the surface characteristics of the gold electrode or the modified electrode. The CV results are shown in Figure 4A. The bare gold electrode presented a pair of obvious redox peaks. Due to the inherent negative carboxyl of GO and BSA (PI 4.7), a redox probe with the same charge was hindered in its approach to the electrode surface because of electrostatic repulsion, which blocked the electron transfer and resulted in a significant decrease in the peak current [27,28,29]. The variation of peak current indicated Nafion–BSA, Nafion–GO or Nafion–GO@BSA was successfully attached to the electrode surface. The BSA amino group was coupled with the GO carboxyl group to form an amide bond, which probably resulted in a decrease of the negative-charge density on the GO surface. Thus, the peak current of Nafion–GO@BSA was higher than that of Nafion–GO and lower than that of Nafion–BSA.

EIS reflected the surface modification of the electrode. As shown in Figure 4B, the semicircle diameter in the high frequency region represents the charge transfer resistance (*R*_ct_), and the line in the low frequency region represents the diffusion process. According to the different modification process of the electrode surface, the electron transfer changed significantly. The semicircle diameter of the bare gold electrode was the smallest (*R*_ct_^bare EG^ 62.7 Ω), implying that the charge transfer was prone to occur. *R*_ct_^GO^ and *R*_ct_^GO@BSA^ for the Nafion–GO-EG and Nafion–GO@BSA-EG, were determined as 1457.0 Ω and 700.8 Ω respectively, thus indirectly indicating that BSA had been successfully coupled with GO. The coverage of the electrode surface (*θ*) by various materials can be calculated by Equation (1) [30],

(1) $$\theta =1-\frac{R_{ct}^{bare}}{R_{ct}}$$

where *θ*^Nafion–GO^ and *θ*^Nafion–GO@BSA^ were determined to be 95.7% and 91.1% respectively. The EIS test results were consistent with the CV results.

We also employed DPVs to further estimate the detection range of the Nafion–GO@BSA-EG electrode for Trp. The bare GE and Nafion–GO-EG lacked chiral recognition sites and so could not recognize Trp enantiomers (Supplementary Figure S3). A satisfactory linear relationship between the logarithmic concentration lg *c* and the percentage of current change (*I* − *I*_0_)/*I*_0_ was obtained using an R^2^ of 0.91 for L-Trp and 0.99 for D-Trp. The LOD was determined to be 10.9 μM, suggesting a detection range from 4 to 500 μM (Supplementary Figure S4).

### 3.3. Response of Sensors to Different Chiral Molecules

**Figure 5:**
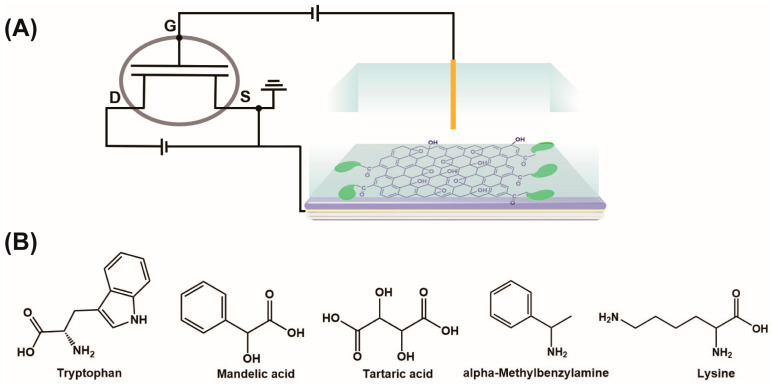
(**A**) Schematic diagram of the Nafion–GO@BSA–EG-MOSFET sensor and (**B**) structures of chiral compounds.

Next, we used the Nafion–GO or Nafion–GO@BSA gold electrode as an EG to structure an EG-MOSFET chiral sensor as shown in Figure 5A, where the MOSFET was meant to magnify the interaction difference between BSA and each isomer to provide highly sensitive chiral resolution. We performed a real-time sensing response for various chiral enantiomers (Figure 5B).

**Figure 6:**
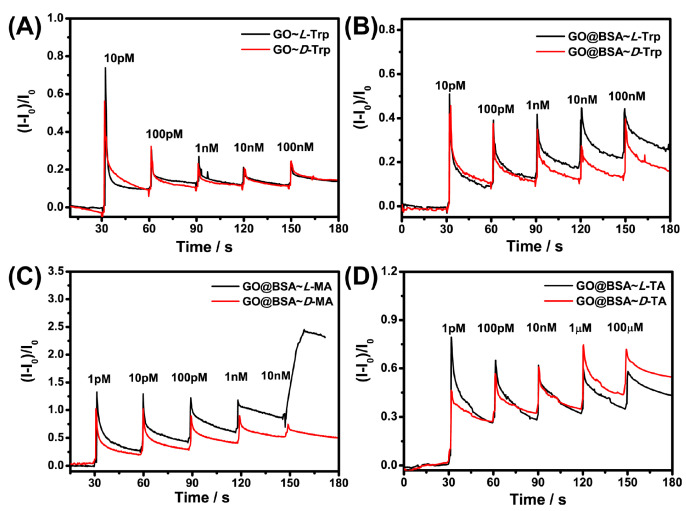
Real time detection curve of Nafion–GO-EG-MOSFET and Nafion–GO@BSA–EG-MOSFET for (**A**,**B**) Trp, (**C**) MA and (**D**) TA enantiomers.

As presented in Figure 6A, the Nafion–GO could not recognize L-Trp or D-Trp enantiomers because it lacked chiral recognition sites. Owing to the introduction of BSA, the Nafion–GO@BSA–EG-MOSFET had good chiral recognition ability for Trp (Figure 6B), TA (Figure 6C) and MA (Figure 6D) as predicted, and realized chiral resolution for all the testing isomers in the range of 1 pM–1 μM. Moreover, when comparing Figure 6C,D, an exciting phenomenon was found: When the analyte carried more negative charge in the solution, the chiral resolution’s lowest concentration gradually increased. Since GO carries more negative charge carboxylic groups, the electrostatic charge prevented the analyte from approaching the chiral sites.

**Figure 7:**
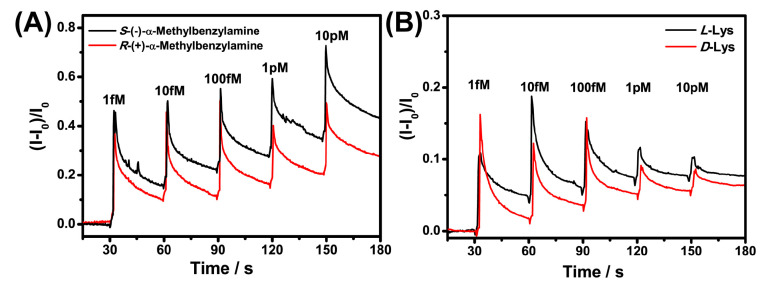
Chiral recognition of positively charged α-Met (**A**) and Lysine (**B**) enantiomers by Nafion–GO@BSA-EG-MOSFET.

To verify this hypothesis, we selected molecules with positive charge (α-Met and Lys) at pH 7 for chiral recognition. As shown in Figure 7, the chiral resolution of α-Met and Lys was achieved at 1 fM, which indicated a positive contribution of electrostatic interaction to the chiral recognition. In addition, the chiral response sensitivity of Nafion–GO@BSA–EG-MOSFET to α-Met containing benzene rings was significantly higher than for the Lys of the alkyl chain. The hydrophobic interaction also played a positive role in protein enantiomer recognition. The real-time response curve demonstrated that the weak enantioselective interactions between BSA and each isomer at such a low concentration can be effectively converted into current signals of different intensities and amplified by the prepared platform to obtain a high chiral sensitivity.

**Figure 8:**
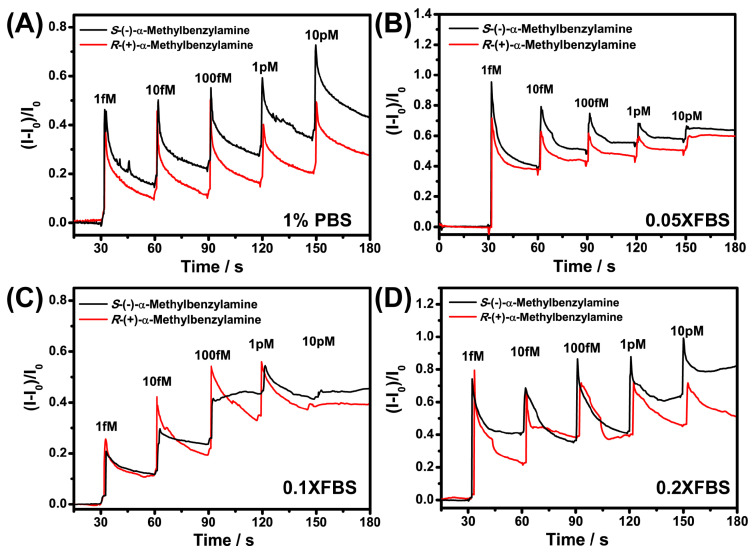
Comparison of chiral recognition ability of the Nafion–GO@BSA–EG-MOSFET platform for α-Met enantiomers in 1% PBS (**A**) and 20-fold (**B**), 10-fold (**C**) and 5-fold (**D**) diluted FBS, respectively.

The anti-interference performance of GO in detecting analytes was noticeable [31,32,33], which suggested that the GO@BSA complex may actually realize detection in blood samples. Inspired by that prospect, we explored the chiral recognition ability of the Nafion–GO@BSA–EG-MOSFET in FBS diluted by a 1% phosphate buffer 20-fold (Figure 8B), 10-fold (Figure 8C) and 5-fold (Figure 8D), respectively. As shown in Figure 8A, in the absence of FBS, the lowest detected concentration of Nafion–GO@BSA–EG-MOSFET to α-Met enantiomer was achieved at the femtomole level, and the sensing platform still maintained good chiral recognition ability with the decrease in the FBS dilution ratio. As shown in Figure 8D, the lowest resolution concentration in 5-fold diluted FBS was realized at the pM level.

**Figure 9:**
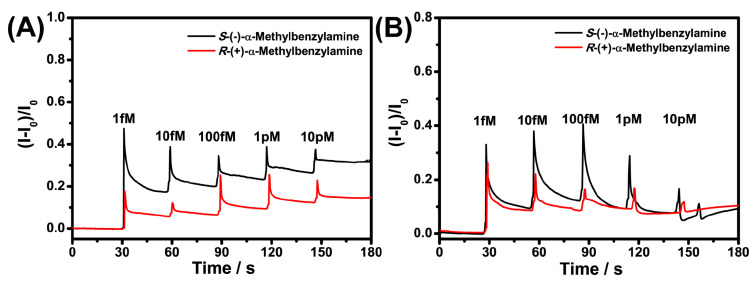
Recognition performance of Nafion–BSA-EG-MOSFET for α-Met enantiomers in 1% PBS (**A**) and 20-fold diluted FBS (**B**).

To further investigate the anti-interference effect of GO, the chiral discrimination of Nafion–BSA–EG-MOSFET to α-Met was studied in 20-fold diluted FBS. As shown in Figure 9A, Nafion–BSA–EG-MOSFET had high sensitivity for α-Met chiral recognition in 1% PBS, but the sensing results in 20-fold diluted FBS showed that the complex detection environment was unfavorable for the chiral detection of BSA (Figure 8B), which led to the decline of sensing ability. The introduction of GO improved the anti-interference ability of the sensor and the detection level in a complex matrix.

### 3.4. Mechanism Investigation

**Figure 10:**
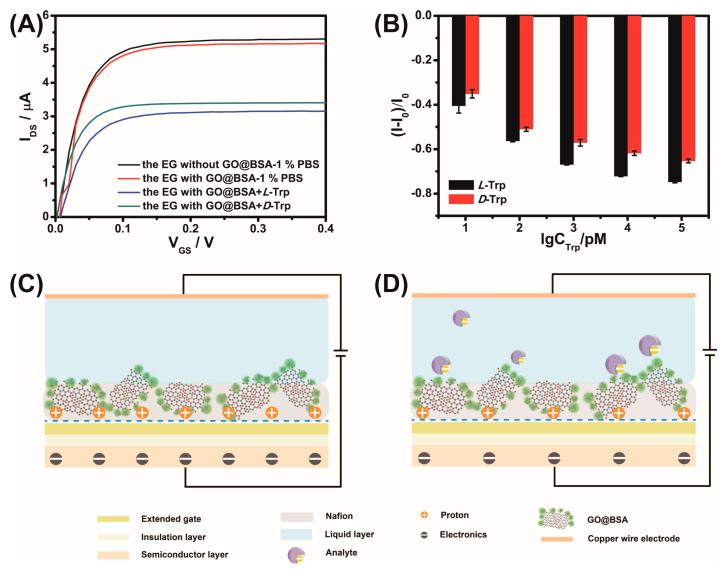
Non-real-time detection of Trp by Nafion–GO@BSA–EG-MOSFET (**A**,**B**) and schematic illustration of chiral recognition mechanism (**C**,**D**).

Taking Trp as a model analyte, the sensing mechanism was explored. The drain current in the saturation region of the n-type MOSFET decreased because of the negative charge of Nafion–GO@BSA at pH 7 as shown in Figure 10A (black line vs. red line). BSA tends to combine with L-Trp [18], which made the current change more obvious (Figure 10A, blue line vs. green line). With the increase of the concentration of the analyte with negative charge, the current in the saturation region of the non-real-time output characteristic curve decreased continuously (Figure 10B). The main reason for this phenomenon was that, under high current density or an ion concentration gradient, the Nafion membrane will transfer water molecules and cations while hindering anions [34]. Hence, the BSA exposed outside the membrane was instrumental in recognizing the negatively charged molecules. As shown in Figure 10C, the polyelectrolytic property of the Nafion membrane meant that many mobile protons existed [35]. The interaction between GO and water molecules, and the subsequent reaction between the water molecules and the GO surface functional groups, led to the production of protons. GO forms an effective fast-transport proton channel at the interface [36], so the biased distribution at the gate is ultimately determined by the proton concentration in the Nafion membrane. When BSA binds to negatively charged D-Trp and L-Trp, the proton concentration in the membrane was neutralized; consequently, fewer electrons were induced in the channel [37], resulting in a decrease in the output current (Figure 10D). The non-real-time current variation of lysine with positive charge was opposite to that of Trp with negative charge (Supplementary Figure S5 vs. S10B), which strongly verified the above inference.

## 4. Conclusions

This work reported the first extended-gate field-effect transistor chiral sensor with a graphene oxide–bovine serum albumin complex. GO@BSA was modified on the extended gate to construct a novel structure Nafion–GO@BSA–EG-MOSFET, which realized the detection of negatively charged molecules (Trp, MA, TA) and positively charged molecules (α-Met, Lys). Electrostatic interaction induced the sensing platform to have ultra-high sensitivity (fM level) to positively charged chiral isomers. The introduction of GO made the sensor maintain a superior ability for chiral recognition in a complex matrix (FBS). The results showed that the sensor has great application potential for chiral sensing and has a certain universality that can realize the rapid and sensitive response of chiral isomers.

## Figures and Tables

**Table 1:** Summary table composition of each substance.

	C	N	O	S
GO	68.7%	1.41%	29.27%	0.62%
GO@BSA	66.09%	9.84%	23.74%	0.32%

## Data Availability

Not applicable.

## Supplementary Materials

The following are available online at https://www.mdpi.com/article/10.3390/s21113921/s1, Figure S1: Optical microscope morphology of Nafion–GO@BSA film on gold surface with doping ratio of 2:1 (A),1:1 (B) and 1:2 (C) (same volume); Figure S2: O1s region of the XPS spectra of (A) GO, (B) GO–COOH, (C) GO@BSA and N1s region of (D) GO@BSA; Figure S3: Differential pulse voltammograms of 5 mM *L*- and *D*-Trp combined with bare gold electrode (A) and Nafion–GO-EG (B) in 0.1 M KCl containing 5 mM [Fe(CN)_6_]^3-/4-^ of pH 7; Figure S4: Differential pulse voltammograms of *L*-Trp (A) or *D*-Trp (B) in the range of 4 μM ~ 500 μM and calibration plot of peak current versus logarithmic concentration of Trp isomers (C); Figure S5: Non-real time detection of *L*-Lys by Nafion–GO@BSA-EG-MOSFET; Table S1: Summary table of peak separation results of O and N elements of each substance.
