# Supplementary material for: Construction and Application of Graphene Oxide-Bovine Serum Albumin Modified Extended Gate Field Effect Transistor Chiral Sensor
Source: Sensors (Basel). 2021 Jun 7;21(11):3921. doi: 10.3390/s21113921 (PMC8201299; doi:10.3390/s21113921)
Supplement: Supplementary file 1 [file sensors-21-03921-s001.zip › sensors-1224175-SI.pdf]

Supplementary Materials

Construction and Application of Graphene Oxide-Bovine Serum Albumin Modified Extended Gate Field Effect Transistor Chiral Sensor

Le Li [1], Xiaofei Ma [1,*], Yin Xiao [2] and Yong Wang [1,*]

[1] Tianjin Key Laboratory of Molecular Optoelectronic Science, Department of Chemistry, School of Science, Tianjin University, Tianjin 300072, China; ll17865181937@163.com
[2] School of Chemical Engineering and Technology, Tianjin Engineering Research Center of Functional Fine Chemicals, Tianjin University, Tianjin 300072, China; xiaoyin@tju.edu.cn
* Correspondence: maxiaofei@tju.edu.cn (X.M.); wangyongtju@tju.edu.cn (Y.W.)

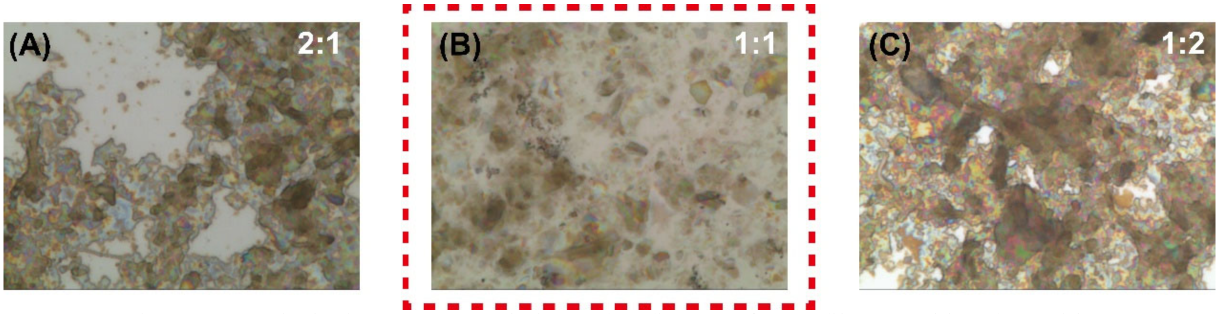

Figure S1. Optical microscope morphology of Nafion–GO@BSA film on gold surface with doping ratio of 2:1 (A),1:1 (B) and 1:2 (C) (same volume).

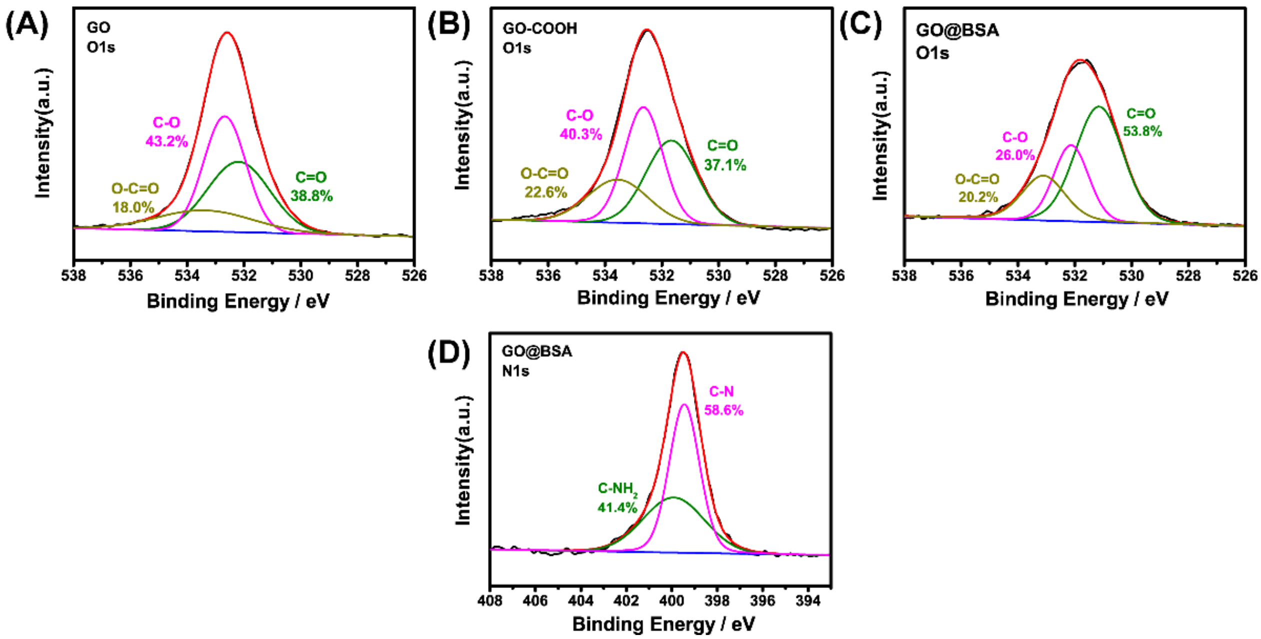

Figure S2. O1s region of the XPS spectra of (A) GO, (B) GO–COOH, (C) GO@BSA and N1s region of (D) GO@BSA

Table S1. Summary table of peak separation results of O and N elements of each substance.

	O			N	
	**C=O**	**C–O**	**O=C–O**	**C–N**	**C–NH_2**
**GO**	**38.8%**	**43.2%**	**18.0%**	–	–
**GO–COOH**	**37.1%**	**40.3%**	**22.6%**	–	–
**GO@BSA**	**53.8%**	**26.0%**	**20.2%**	**58.6%**	**41.4%**

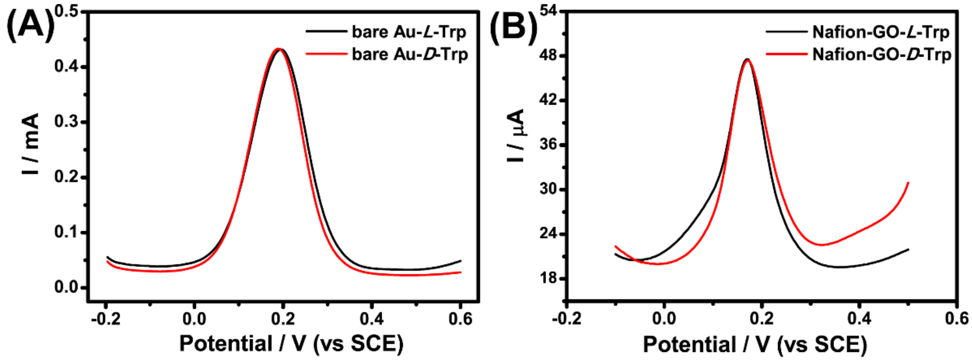

Figure S3. Differential pulse voltammograms of 5 mM *L*- and *D*-Trp combined with bare gold electrode (A) and Nafion–GO-EG (B) in 0.1 M KCl containing 5 mM $[Fe(CN)_6]^{3-/4-}$ of pH 7.

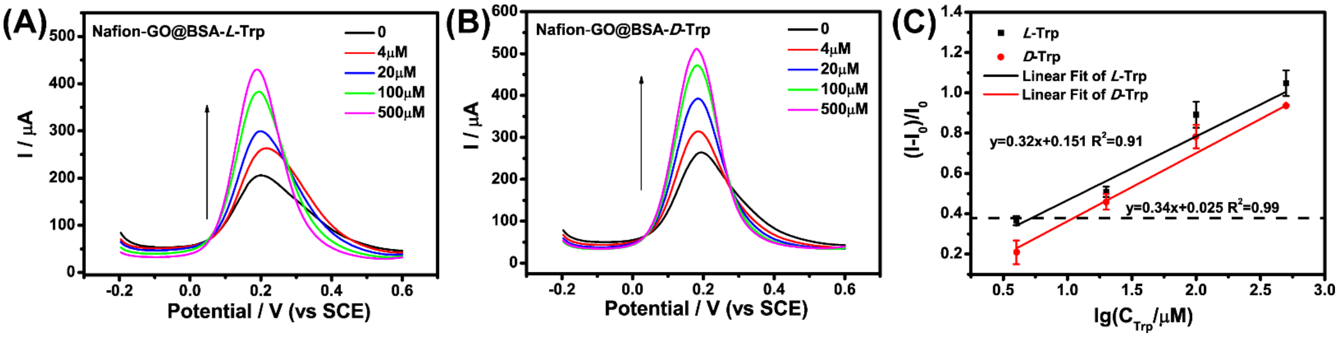

Figure S4. Differential pulse voltammograms of *L*-Trp (A) or *D*-Trp (B) in the range of 4 μM ~ 500 μM and calibration plot of peak current versus logarithmic concentration of Trp isomers (C).

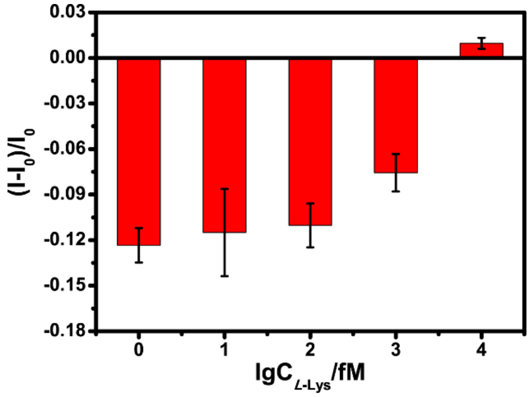

Figure S5. Non-real time detection of *L*-Lys by Nafion–GO@BSA-EG-MOSFET.
